# Supplementary material for: HPV Infections—Classification, Pathogenesis, and Potential New Therapies
Source: Int J Mol Sci. 2024 Jul 11;25(14):7616. doi: 10.3390/ijms25147616 (PMC11277246; doi:10.3390/ijms25147616)
Supplement: Supplementary file 1 [file ijms-25-07616-s001.zip › ijms-3028618-supplementary.pdf]

Table S1. Classification of HPV

HPVs classified as oncogenic and probably oncogenic are not underlined; those which are possibly oncogenic are underlined in green. In green underlining are HPV types classified as possibly oncogenic in patients with EV [147]. Links to Genbank are placed in {}. ICTV—International Committee on Taxonomy of Viruses, TB—Taxonomy Browser, IRHC—International Human Papillomavirus Reference Center, PaVe—The Papilloma Virus Episteme.

Viruses (superkingdom); Monodnaviria (clade); Shotokuvirae (kingdom); Cossaviricota (phylum); Papovaviricetes (class); Zurhausenvirales (order); Papillomaviridae (family); Firstpapillomavirinae (subfamily);

**genus**	**Alphapapillomavirus**
species	**Alphapapillomavirus 1** [16-18]
type	HPV32 { X74475, OP712042 }; HPV42 {M73236, KU298897}
species	**Alphapapillomavirus 2** [17, 19- 23,]
type	HPV3 {X74462}; HPV10 {X74465,NC_001576}; HPV28 {U31783}; HPV29 {U31784}; HPV77 {Y15175}; HPV78 {KC138720, AB793779}; HPV94 {AJ620211}; HPV117 {GQ246950}; HPV125 {FN547152}; HPV160 {AB745694};
	In addition, the TB[13] database includes HPVXS2 (isolate GX-70) {OL817334}.
species	**Alphapapillomavirus 3** [16, 24- 32,]
type	HPV61 sublineage A1, A2, {U31793, KF436853} lineage B, C {KF436856, KF436858}; HPV62 {LR862034}; HPV72 {X94164}; HPV81 {AJ620209}; HPV83 {AF151983}; HPV84 {LR861944}; HPV86 {OP712081}; HPV87 {OP711973}; HPV89 {OP971058}; HPV102 {KU298947}; HPV114 {GQ244463}
species	**Alphapapillomavirus 4 [**17, 33-35,]
type	HPV2 {NC_001352}, lineage 2a {X55964} and 2c {AH004565}; HPV27 lineage b {AB211993}; HPV57 {MK463925} lineage b {U37537}, lineage c {AB361563}
species	**Alphapapillomavirus 5** [17, 25, 36-38]
type	HPV26 lineage A {X74472};
	HPV51 sublineage A1 to A4, B1, B2 {M62877, KF436870, KF436873, KF436875, KF436883, KF436886};
	HPV69 sublineage A1 to A4 {AB027020, KF436859, KF436861, KF436863};
	HPV82 sublineage A1 to A3, B1, B2, C1 to C5) {AB027021, KF436787, KF436793, KF436794, KF444055, AF293961, KF436800, KF436801, KF436802, KF436803}
species	**Alphapapillomavirus 6** [17, 25, 39, 40]
type	HPV30 sublineage A1 to A3 {X74474, KF436842, KF436844} and lineage B{KF436850};
	HPV53 lineage A, B, C, {X74482, KF436818, EF546477} and sublineage D1 to D4 { EF546482, KF436823, GQ472849, EF546479};
	HPV56 sublineage A1, A2 {X74483, EF177179} and lineage B {EF177176};
	HPV66 lineage A {U31794 } and sublineage B1, B2) { EF177188, EF177187}
species	**Alphapapillomavirus 7** [17, 25, 38, 41-51]
type	HPV18 sublineage A1 and A2 (Asian-Amerindian), A3 to A5 (European), B1 to B3 (African) {AY262282, EF202146, EF202147, EF202151, GQ180787, EF202155, KC470225, EF202152} and lineage C (African) {KC470229};
	HPV39 sublineage A1, A2 { M62849, KC470239} and lineage B {KC470247};
	HPV45 sublineage A1 to A3, B1, B2 {X74479, EF202157, KC470256, EF202161, EF202164};
	HPV59 sublineage A1 to A3, B {X77858, KC470261, KC470263} and lineage B{KC470264};
	HPV68 lineage a {LR861886}, sublineage A1, A2 {X67161 partial, KC470269}, lineage B and b {KC470270, FR751039}, sublineage C1, C2 {FR751039, KC470274}, sublineage D1, D2 {KC470275, KC470276}, lineage E {KC470277} and sublineage F1, F2 { KC470279, KC470281};
	HPV70 lineage A and B {U21941, KC470287}; HPV85 lineage A {AF131950}; HPV97 lineage A {EF202168}
species	**Alphapapillomavirus 8** [16, 17, 28]
type	HPV7 {MK463913}; HPV40 {KU298895}; HPV43 {OP712100}; HPV91 {OP971097}
species	**Alphapapillomavirus 9** [17, 25, 42, 52-68]
type	HPV16 sublineage A1 to A3 (European){ K02718, AF536179, HQ644236}, A4 (Asian) {AF534061}, B1 (African-1, Afr1a) {AF536180} B2 African-1, Afr1b) {HQ644298}, B3 and B4 { KU053915, KU053914}, C1 (African-2, Afr2a) AF472509, C2, C3, C4 { HQ644244, KU053920, KU053925}, D1 (North American, NA1){ HQ644257}, D2 (Asian-American, AA2){ AY686579}, D3 (Asian-American AA1){ AF402678} and D4{KU053931};

	HPV31 sublineage A1, A2, B1, B2, C1 to C4 {J04353, HQ537675, HQ537676, HQ537680, HQ537682, HQ537684, HQ537685, MT752407};
	HPV33 sublineage A1 to A3, B1, C1 {M12732, HQ537698, EU918766, HQ537705, KF436865};
	HPV35 sublineage A1, A2 {X74477, HQ537727};
	HPV52 A1, A2, B1, B2, B3, C1, C2, D1, E1 {X74481, HQ537739, HQ537740, HQ537743, HQ537744, HQ537746, HQ537748};
	HPV58 sublineage A1 to A3, B1, B2, C1, D1, D2 {D90400, HQ537752, HQ537758, HQ537762, HQ537764, HQ537774, HQ537768, HQ537770};
	HPV67 sublineage A1, A2, B1) {D21208, HQ537780, HQ537783}
species	**Alphapapillomavirus 10** [16, 69- 84]
type	HPV6 lineage A {X00203}, 6a (classified as sublineage B3) {L41216}, 6 b (classified as lineage A) {NC_001355}, 6c {M26656 partial sequence}, 6e, 6vc (classified as sublineage B1) {AF092932} and sublineage B1 {FR751337, AF092932}, B2 {FR751328}, B3 {L41216}, B4 {JM169762}, B5 {JM169759};
	HPV11 sublineage A1 {M14119}, A2 {FN907962}, A3 (isolate C185), A4 (isolate LT4) and lineage B (isolate SA3408); HPV13 {MT068446}; HPV44 {KU298900}; HPV74 {LR862050}
	In addition, the TB database[13] includes HPV55 {LR861883}
species	**Alphapapillomavirus 11** [17, 25, 85]
type	HPV34 sublineage A1, A2 {X74476, KF436808}, lineage B {KF436810} and sublineage C1, C2 { KF436812, KF436816};
	HPV73 sublineage A1, A2 {X94165, KF436829} and lineage B {KF436834};
	In addition, the TB database[13] includes HPV177 {KR816168}
species	**Alphapapillomavirus 13** [25, 38]
type	HPV54 lineage A, B, C) {U37488, AF436129, KF436894}
species	**Alphapapillomavirus 14** [28, 29, 38]
type	HPV71 {AB040456}; HPV90 {NC_004104}; HPV106 {DQ080082}
	**genus Betapapillomavirus**
species	**Betapapillomavirus 1** [17, 86-96]
type	HPV5 {JN211194} lineage b {D90252}; HPV8 {M12737}; HPV12 {X74466}; HPV14 {X74467} lineage D {X74467}; HPV19 {X74470}; HPV20 {U31778}; HPV21 {U31779}; HPV24 {U31782}; HPV25 {X74471}; HPV36 {U31785}; HPV47 {M32305}; HPV93 {AY382778}; HPV98 {FM955837}; HPV99 {FM955838}; HPV105 {FM955841}; HPV118 {GQ246951}; HPV124 {GQ845446}; HPV143 {HM999995}; HPV152 {JF304768}
	In addition, the [13] database includes HPVRTRX7{U85660}; HPVV001/Slovenia/2010 {FR822732 partial sequence}
	In addition, the [14] database includes HPV195 {KR816182}; HPV196 {KR816183}; HPV206 {U85660} in ICTV [12] appear as unclassified Betapapillomavirus HPVmRTRX7;
species	**Betapapillomavirus 2** [17, 19, 89, 97-107]
type	HPV9 {NC_001596}; HPV15 {X74468}; HPV17 {JN211195}; HPV22 {U31780}; HPV23 {U31781}; HPV37 {U31786}; HPV38 {U31787}, lineage b {DQ090005} ; HPV80 {Y15176}; HPV100 {FM955839}; HPV104 {FM955840}; HPV107 {EF422221}; HPV110 {EU410348}; HPV111 {EU410349}; HPV113 {FM955842}; HPV120 {FN598907}; HPV122 {GQ845444}; HPV145 {HM999997}; HPV151 {FN677756}; HPV159 {HE963025}; HPV174 {HF930491};
	In addition, the [14] database includes HPV182 {KR816170}; HPV198 {MG921179}; HPV209 {KY242583}; HPV227 {MK080568}
	In addition, the [13] database includes HPVFA75/KI88-03
species	**Betapapillomavirus 3** [17, 109]
type	HPV49 {NC_001591}; HPV75 {Y15173}; HPV76 {Y15174}; HPV115 {FJ947080}
species	**Betapapillomavirus 4** [110]
type	HPV92 {NC_004500}
	**Betapapillomavirus 5** [92, 101, 111]
	HPV96 {AY382779}; HPV150 {FN677755}
	In addition, the[13] and [14]] databases include HPV185 {KR816172}
**genus**	**Gammapapillomavirus**
species	**Gammapapillomavirus 1** [112-114]
type	HPV4 {NC_001457; X70827}; HPV65 {X70829}; HPV95 {AJ620210}; HPV173 {KF006400}; HPV205 {KT698167}
species	**Gammapapillomavirus 2** [115, 116]
type	HPV48 {NC_001690}; HPV200 {KP692114}
species	**Gammapapillomavirus 3** [117]
type	HPV50 {NC_001691};

	In addition, the [13] and [14]] databases include HPV188 {KR816175}
species	**Gammapapillomavirus 4**
type	HPV60 {NC_001693} [118]
species	**Gammapapillomavirus 5**
type	HPV88 {NC_010329} [119]
species	**Gammapapillomavirus 6** [120-123]
type	HPV101 {OP712017}; HPV103 {LR861918}; HPV108 {NC_012213}
	In addition, the [14] database includes HPV214 {MF509819}; HPV226 {MG813996}
species	**Gammapapillomavirus 7** [32, 94, 95, 124-127]
type	HPV109 {NC_012485}; HPV123 {GQ845445}; HPV134 {NC_014956}; HPV138 {HM999990}; HPV139 {HM999991}; HPV149 {GU117629}; HPV155{JF906559}; HPV170 {JX413110}
	In addition, the [14] and [13] databases include HPV186 {KR816173}; HPV189 {KR816176}; HPV193 {KR816180};
	Furthermore, the [14] database includes HPV203 {MG921180}; HPV225 {MG520499}; HPV229 {MW535770}
species	**Gammapapillomavirus 8 [**32, 94, 122, 126, 128]
type	HPV112 {NC_012486}; HPV119 {GQ845441}; HPV147 {HM999999}; HPV164 {JX413106}; HPV168 {KC862317};
	Furthermore, the [14] and [13] databases include HPV176 (KR816167).
	a In addition, the [14] database includes HPV211 {MF509816}; HPV224 {MF356498} which in ICTV [12] appears as unclassified Betapapillomavirus HPVmICB1.
species	**Gammapapillomavirus 9** [122, 125, 129]
type	HPV116 {FJ804072}; HPV129 {NC_014953};
	In addition, the [14] database includes HPV 215 {MF509820}; 216 {MF509821}
species	**Gammapapillomavirus 10** [94, 125, 130, 131]
type	HPV121 {NC_014185}; HPV130 {GU117630}; HPV133 {GU117633}; HPV142 {HM999994}; HPV180 {KC108722};
	In addition, the [14] and [13] databases include HPV191 {KR816178};
	Furthermore, the [14] database includes HPV221 {MH172378}; HPV231 {OP577477}
species	**Gammapapillomavirus 11** [95, 113, 116, 126, 132, 133]
type	HPV126 {NC_016157}; HPV136 {NC_017994}; HPV140 {NC_017996}; HPV141 {HM999993}; HPV154 {NC_021483}; HPV169 {JX413105}; HPV171 {KF006398}; HPV202 {KP692116}
	In addition, the [14] and [13] databases include HPV181 {KR816169} a
	Furthermore, the [13] database includes HPV230 {OQ915151}
species	**Gammapapillomavirus 12** [114, 125, 126, 134-136]
type	HPV127 {NC_014469}; HPV132 {NC_014955}; HPV148 {GU129016}; HPV157 {KT698166}; HPV158 {KT698168}; HPV165 {JX444072}; HPV199 {KJ913662};
	In addition, the [14] and [13] databases include HPV210 {MH460956}
species	**Gammapapillomavirus 13** [122, 125, 131, 137]
type	HPV128 {NC_014952}; HPV153 {JN171845};
	In addition, the [14] database includes HPV213 {MF509818}; HPV219 {MH172376}
species	**Gammapapillomavirus 14** [125]
type	HPV131 {NC_014954}
species	**Gammapapillomavirus 15** [95, 113, 138, 139]
type	HPV135 {NC_017993}; HPV146 {HM999998}; HPV179 {NC_022095};
	In addition, the [14] and [13] databases include HPV192 {KR816179} a
	In addition, the [14] database includes HPV230 {OQ915151}
species	**Gammapapillomavirus 16** [95]
type	HPV137 {NC_017995}
species	**Gammapapillomavirus 17** [95, 122, 131]
type	HPV144 {NC_017997};
	In addition, the [14] database includes HPV212 {MF509817}; HPV220 {MH172377}
species	**Gammapapillomavirus 18** [140]
type	HPV156 {NC_033781}
species	**Gammapapillomavirus 19** [126, 131]

type	HPV161 {NC_038522}; HPV162 {JX413108}; HPV166{NC_019023};<br>In addition, the [14] database includes HPV222 {MH172379}
species	**Gammapapillomavirus 20** [126]
type	HPV163 {NC_028125};<br>In addition, the [14] and [13] databases include HPV 183 {KR816171} a<br>Furthermore, the [14] database includes HPV194 {KR816181}
species	**Gammapapillomavirus 21** [128]
type	HPV167 {NC_022892}
species	**Gammapapillomavirus 22** [113]
type	HPV172 {NC_038523};<br>In addition, the [14] database includes HPV223 {MG063749}
species	**Gammapapillomavirus 23** [130]
type	HPV175 {NC_038524}
species	**Gammapapillomavirus 24** [141, 142]
type	HPV178 {NC_023891}; HPV197{KM085343};<br>In addition, the [14] database includes HPV190 {KR816177}; HPV208 {MK645901}
species	**Gammapapillomavirus 25** [138]
type	HPV184 {NC_038914; HG530535}
species	**Gammapapillomavirus 26**
type	HPV187 {NC_039086; KR816174};
species	**Gammapapillomavirus 27** [116]
type	HPV201 {NC_027528; KP692115};<br>In addition, the [14] database includes HPV228 {ON482334}
**genus**	**Mupapillomavirus**
species	**Mupapillomavirus 1** [143]
type	HPV1 {V01116}<br>In the [13] database it appears under the name HPV1a {V01116}
species	**Mupapillomavirus 2** [112]
type	HPV63 {X70828}
species	**Mupapillomavirus 3** [144, 145]
type	HPV204 {KP769769}
**genus**	**Nupapillomavirus**
species	**Nupapillomavirus** 1 **[146]**
type	HPV41 { X56147}

Table S2. HPV species, genome and proteins.

Abreviations: L or S- lineage or sublineage; Alpha-, Beta-, Gamma-, Mu- and Nu- Alphapapillomavirus, Betapapillomavirus, Gammapapillomavirus, Mupapillomavirus, Nupapillomavirus respectively; HPV46, 64, 79, 217, 218 are not present because they have been reclassified to HPV20, 34, 91, 182, 189 respectively; * Determination of species by International Human Papillomavirus Reference Center [14], and according to the TB database [13], species undetermined; ** type HPV1, 6 lineage A, 6 sublineage B1,6 sublineage B3, 206, 223, 224, 225, 226, 227,231 appear in the TB and GenBank database as types 1a, 6b, 6vc, 6a, RTRX7, MTS3, ICB1, MTS4, 16031680A, ICB2, CDCHPVTL_S18 respectively; 1partial sequences GenBank acc. No. P06428.1, P06430.1, P06420.1, P06422.1, P06425.1, P0619.1, P0617.1; () the alternative size of the protein is given; + the size of two proteins E5a +E5b is given

HPV	L or S	Species	Genome (bp)	E6 (aa)	E7 (aa)	E1 (aa)	E2 (aa)	E4 (aa)	E5 (aa)	L2 (aa)	L1 (aa)	GenBank acc. no.
1**		Mu-1	7815	140	93					507	508	V01116
2		Alpha-4	7860	159	92	643	391	132		524	510	NC_001252
3		Alpha-2	7820	152 (148)		659	383			473 (532)	504	X74462
4		Gamma-1	7353	140	100	599	402	181		521	516	X70827
5		Beta-1	7746	157	103	606	514			518	516	JN211194
5b		Beta-1	7779	157	103	606	514	245	168	518	525	D90252
6**	A	Alpha-10	7902	150	98	649	368	109	91+72	459	500	X00203
6**	B1	Alpha-10	8031	150	98	649	368	109	91+72	459	500	FR751337
6	B2	Alpha-10	8031	150	98	649	368	109	91+72	459	500	FR751328
6**	B3	Alpha-10	8010	150	98	649	368	99	91+72	459	500	L41216
7		Alpha-8	8037	154	111	646	375	119		456	505	MK463913
8		Beta-1	7654	155	103	603	498	222		518	514	M12737[!]
9		Beta-2	7434	148 (141)	93	605	461			533	507	NC_001596
10		Alpha-2	7919	148	86	681	376			470	531 (503)	NC_001576
	A1	Alpha-10	7931	150	98	649	367	108	91+74	455	501	M14119
		Alpha-10	7934	150	98	649	367	108	91+74	455	501	FN907962
12		Beta-1	7673	157	103	604	494			518	517	X74466
13		Alpha-10	7831	150	98	646	377	118	91	463	499	MT068446
14		Beta-1	7439			605	483			519	518	X74467
15		Beta-2	7412	141	93	602	282			533	507	X74468
16	A1	Alpha-9	7904	158	98	649	365	95	78	473	531	KO2718
16	A2	Alpha-9	7904	158	98	649	365	95	78	473	531	AF536179
16	A3	Alpha-9	7905	151	98	649	365	95	83	473	531	HQ644236
16	A4	Alpha-9	7905	158	98	649	365	95	83	473	531	AF53406
16	B1	Alpha-9	7906	158	98	649	365	95	83	473	531	AF536180
16	B2	Alpha-9	7912	151	98	649	365	95	83	473	531	HQ644298
16	B3	Alpha-9	7909	151	98	649	365	95	83	473	505	KU053915
16	B4	Alpha-9	7907	151	98	649	365	95	83	473	505	KU053914
16	C1	Alpha-9	7904	158	98	649	365	95	83	473	531	AF472509
16	C2	Alpha-9	7904	151	98	649	365	95	83	473	531	HQ644244
16	C3	Alpha-9	7906	151	98	649	365	95	83	473	531	KU053920
16	C4	Alpha-9	7904	151	98	649	365	95	83	473	505	KU053925
16	D1	Alpha-9	7885	151	98	649	365	95	83	473	531	HQ644257
16	D2	Alpha-9	7906	158	98	649	365	95	83	473	531	QV15321
16	D3	Alpha-9	7906	158	98	649	365	95	83	473	531	QV00995
16	D4	Alpha-9	7909	151	98	649	365	95	83	473	505	KU053931
17		Beta-2	7426	141	95	609	452	196		536	507	JN211195
18	A1	Alpha-7	7857	158	105	657	365	88	73	462	568	AY262282
18	A2	Alpha-7	7857	158	105	657	365	88	73	462	568	EF202146
18	A3	Alpha-7	7857	158	105	657	365	88	73	462	568	EF202147
18	A4	Alpha-7	7857	158	105	657	365	88	73	462	568	EF202151
18	A5	Alpha-7	7844	158	105	657	363	86	73	462	568	GQ180787
18	B1	Alpha-7	7824	158	105	657	363	86	73	462	568	EF202155
18	B2	Alpha-7	7824	158	105	657	363	86	73	462	568	KC470225
18	B3	Alpha-7	7844	158	105	657	363	86	73	462	568	EF202152
18	C	Alpha-7	7837	158	105	657	363	86	73	462	568	KC470229
19		Beta-1	7685	157	102	604	493			520	546	X74470
20		Beta-1	7757	165	102	605	497	228		518	516	U31778
21		Beta-1	7779	168	101	603	503	326		520	518	U31779

22		Beta-2	7368	165	100	608	436	193		524	506	U31780
23		Beta-2	7324	157	97	607	431	242		519	507	U31781
24		Beta-1	7452	140	96	607	467	223		523	512	U31782
25		Beta-1	7714	161	102	604	502			520	517	X74471
26		Alpha-5	7855	150	104	638	375			472	503	X74472
27		Alpha-4	7831	159	92	643	388	129		464	608	AB211993
28		Alpha-2	7959	146	87	662	376	102		473	502	U31783
29		Alpha-2	7916	148	90	660	338	115		473	503	U31784
30	A1	Alpha-6	7852	153	105	631	378			463	508	X74474
30	A2	Alpha-6	7843	153	105	631	375	119		463	501	KF436842
30	A3	Alpha-6	7881	153	105	631	377	121		463	501	KF436844
30	B	Alpha-6	7881	153	105	631	377	121		463	501	KF436850
31	A1	Alpha-9	7912	149	98	629	372	102	84	466	504	J04353
31	A2	Alpha-9	7945	149	98	629	372	102	84	466	504	HQ537675
31	B1	Alpha-9	7898	149	98	629	372	102	84	466	504	HQ537676
31	B2	Alpha-9	7886	149	98	629	372	102	84	466	504	HQ537680
31	C1	Alpha-9	7878	149	98	629	372	102	84	466	504	HQ537682
31	C2	Alpha-9	7878	149	98	629	372	102	84	466	504	HQ537684
31	C3	Alpha-9	7878	149	98	629	372	102	84	466	504	HQ537685
31	C4	Alpha-9	7912	149	98	629	372	103	84	466	502	MT752407
32		Alpha-1	7960	142	104	643	394	113	42	476	503	OP712042
33	A1	Alpha-9	7909	149	97	644	353	83	75	467	499	M12732
33	A2	Alpha-9	7831	149	97	644	353	83	75	467	499	HQ537698
33	A3	Alpha-9	7833	149	97	644	353	83	75	467	499	EU918766
33	B	Alpha-9	7832	149	97	644	353	83	75	467	499	HQ537705
33	C	Alpha-9	7820	149	97	644	353	83	75	466	499	KF436865
34	A1	Alpha-11	7723	148	97	647	345			472	528	X74476
34	A2	Alpha-11	7770	148	97	647	345		74	472	502	KF436808
34	B	Alpha-11	7765	148	97	647	345		74	472	502	KF436810
34	C1	Alpha-11	7788	148	97	647	345		74	473	502	KF436812
34	C2	Alpha-11	7788	148	97	647	345		74	473	502	KF436816
35	A1	Alpha-9	7879	149	99	637				469	502	X74477
35	A2	Alpha-9	7879	149	99	637	367	96	83	469	502	HQ537727
36		Beta-1	7722	157	102	604	509	214		518	516	U31785
37		Beta-2	7421	141	94	609	454	207		534	507	U31786
38		Beta-2	7400	141	100	604	441	189		527	510	U31787
39	A1	Alpha-7	7833	158	109	647	370	94	72	470	505	M62849
39	A2	Alpha-7	7860	158	109	656	379	91	72	470	505	KC470239
39	B	Alpha-7	7833	158	109	647	370	91	72	470	505	KC470247
40		Alpha-8	7890	154	111	647	365	109	43	467	505	KU298895
41		Nu-1	7614	156	114	614	387	101	78	554	583	NC_001354
42		Alpha-1	7919	150	93	643	398	120		477	502	KU298897
43		Alpha-8	7968	155	99	655	371	96	44	463	503	OP712100
44		Alpha-10	7780	150	97	643	378	138	92+43	460	500	KU298900
45	A1	Alpha-7	7858	158	106	643	368			463	539	X74479
45	A2	Alpha-7	7858	158	106	643	368	90	73	463	536	EF202157
45	A3	Alpha-7	7841	158	106	643	368			463	536	KC470256
45	B1	Alpha-7	7849	158	106	643	368	90	73	463	536	EF202161
45	B2	Alpha-7	7849	158	106	643	368	90	73	463	536	EF202164
47		Beta-1	7726	156	103	605	506	304		518	514	M32305
48		Gamma-2	7100	142	93	593	396	133		502	513	NC_001690
49		Beta-3	7560	138	103	609	488			521	509	NC_001591
50		Gamma-3	7184	141	93	599	396	146		506	515	NC_001691
51	A2	Alpha-5	7813	151	101	634	358	87		469	504	KF436870
51	A3	Alpha-5	7814	151	101	634	358	87		469	504	KF436873
51	A4	Alpha-5	7811	151	101	634	358	87		469	504	KF436875
51	B1	Alpha-5	7815	151	101	634	358	87		469	504	KF436883
51	B2	Alpha-5	7814	151	101	634	358	87		469	504	KF436886

52	A1	Alpha-9	7942	148	99	647	368			466	529	X74481
52	A2	Alpha-9	7933	148	99	647	368	97	75	466	529	HQ537739
52	B1	Alpha-9	7962	148	99	647	368	97	75	466	529	HQ537740
52	B2	Alpha-9	7960	148	99	647	368	97	75	466	529	HQ537743
52	C1	Alpha-9	7974	148	99	647	368	97	75	466	529	HQ537744
52	C2	Alpha-9	7962	148	99	647	368	97	75	466	527	HQ537746
52	D	Alpha-9	7937	148	99	647	368	97	75	466	530	HQ537748
53	A	Alpha-6	7856	154	105		384			463	499	X74482
53	B	Alpha-6	7881	154	105	636	384	150	85	463	499	KF436818
53	C	Alpha-6	7863	154	105	636	384	150		463	499	EF546477
53	D1	Alpha-6	7863	154	105	636	384	150		463	499	EF546482
53	D2	Alpha-6	7863	154	105	636	384	150	85	463	499	KF436823
53	D3	Alpha-6	7863	154	105	636	384			463	499	GQ472849
53	D4	Alpha-6	7863	154	105	636	384	150		463	499	EF546479
54	A	Alpha-13	7759	144	95	633	367	134		470	497	U37488
54	B	Alpha-13	7717	174	95	634	366	133		470	525	AF436129
54	C	Alpha-13	7708	174	95	635	366	133		470	497	KF436894
55		Alpha-10	7822	150	97	644	378	144		460		LR861883
56	A1	Alpha-6	7844	155	105		310			464	534	X74483
56	A2	Alpha-6	7790	155	105	636	371	135		464	534	EF177179
56	B	Alpha-6	7866	155	105	636	371	135		464	534	EF177176
57		Alpha-4	7848	148	92	643	379	120		465	510	MK463925
58	A1	Alpha-9	7824	149	98	644	358	91	76	472	524	D90400
58	A2	Alpha-9	7824	149	98	644	358	91	76	472	524	HQ537752
58	A3	Alpha-9	7836	149	98	644	358	91	76	472	524	HQ537758
58	B1	Alpha-9	7823	149	98	644	358	91	76	472	524	HQ537762
58	B2	Alpha-9	7825	149	98	644	358	91	76	472	524	HQ537764
58	C	Alpha-9	7820	149	98	644	358	91	76	472	524	HQ537774
58	D1	Alpha-9	7814	149	98	644	357	90	76	472	524	HQ537768
58	D2	Alpha-9	7817	149	98	644	358	91	76	472	524	HQ537770
59	A1	Alpha-7	7896	160	107	644	370	115	73	464	508	X77858
59	A2	Alpha-7	7898	160	107	644	370	115	73	464	508	KC470261
59	A3	Alpha-7	7897	160	107	644	370	115	73	464	508	KC470263
59	B	Alpha-7	7898	160	107	644	370	115	73	464	508	KC470264
60		Gamma-4	7313	142	96	610	404	207		525	508	NC_001693
61	A1	Alpha-3	7989	146	95	652	382	105		457	505	U31793
61	A2	Alpha-3	7989	146	95	652	382	104		459	505	KF436853
61	B	Alpha-3	8015	146	95	653	382	104		459	505	KF436856
61	C	Alpha-3	8027	146	95	653	382	104		459	505	KF436858
62		Alpha-3	8092	148	96	652	379	104	46	475	503	LR862034
63		Mu-2	7348	141	88	618	398	155		504	507	NC_001458
65		Gamma-1	7308	140	98	598	402	213		518	516	X70829
66		Alpha-6	7824	155	105	630	369	174		464	503	U31794
67	A1	Alpha-9	7801	149	99	636	368	97	73	465	539	D21208
67	A2	Alpha-9	7803	149	99	636	368	97	73	465	539	HQ537780
67	B	Alpha-9	7809	149	99	636	370	99	73	465	539	HQ537783
68	A2	Alpha-7	7822	158	110	640	370		73	469	505	KC470269
68	B	Alpha-7	7814	158	110	640	370		73	469	505	KC470270
68	C1	Alpha-7	7836	158	110	640	370	94	73	469	505	FR751039
68	C2	Alpha-7	7836	158	110	640	370		73	469	505	KC470274
68	D1	Alpha-7	7830	158	110	640	370		73	469	505	KC470275
68	D2	Alpha-7	7830	158	110	640	370		73	469	505	KC470276
68	E	Alpha-7	7830	158	110	640	370		73	469	505	KC470277
68	F1	Alpha-7	7828	158	110	640	370		73	469	505	KC470279
68	F2	Alpha-7	7838	158	110	640	370		73	469	505	KC470281
69	A1	Alpha-5	7700	151	104	634	368	101	98	467	507	AB027020
69	A2	Alpha-5	7705	151	104	634	368	101	98	467	508	KF436859
69	A3	Alpha-5	7705	151	104	634	368	101	98	467	508	KF436861

69	A4	Alpha-5	7705	151	104	634	368	101	98	467	508	KF436863
70	A	Alpha-7	7905	158	109	652	360	84	78	466	504	U21941
70	B	Alpha-7	7922	158	109	652	360	81	78	466	506	KC470287
71		Alpha-14	8017	156	105	642	384	106	47	504	509	NC_039089
72		Alpha-3	7988	148	100	650	384	106		466	534	X94164
73	A1	Alpha-11	7700	148	97	650	350			475	503	X94165
73	A2	Alpha-11	7700	148	97	650	350	78	74	475	503	KF436829
73	B	Alpha-11	7897	148	97	650	349	77	74	475	503	KF436834
74		Alpha-10	7902	150	95	640	381	141	91	464	527	LR862050
75		Beta-3	7537	138	101	609	469	215		521	509	Y15173
76		Beta-3	7549	138	101	609	478	224		521	509	Y15174
77		Alpha-2	7887	148	90	661	373	136		519	565	Y15175
78		Alpha-2	7805	148	87	660	378	101		475	504	AB793779
80		Beta-2	7427	141	93	605	444	235		533	507	Y15176
81		Alpha-3	8070	154	98	650	378	102		472	532 (504)	AJ620209
82	A1	Alpha-5	7871	151	100	642	359	88	84	459	503	AB027021
82	A2	Alpha-5	7872	151	100	642	359	88	83	473	534	KF436787
82	A3	Alpha-5	7870	151	100	642	359	88	83	473	534	KF436793
82	B1	Alpha-5	7874	151	100	642	359	88	83	473	534	KF436794
82	B2	Alpha-5	7872	151	100	642	359	88	83	473	534	KF444055
82	C1	Alpha-5	7904	151	100	641	359	88	82	473	503	AF293961
82	C2	Alpha-5	7904	151	100	641	359	88	82	473	534	KF436800
82	C3	Alpha-5	7904	151	100	641	359	88	82	473	534	KF436801
82	C4	Alpha-5	7904	151	100	641	359	88	82	473	534	KF436802
82	C5	Alpha-5	7908	151	100	641	359	88	82	473	534	KF436803
83		Alpha-3	8104	147	97	651	380	113		471	503	AF151983
84		Alpha-3	7956	148	96	651	377	103		474	503	LR861944
85		Alpha-7	7812	157	108	648	377	95	73	467	504	AF131950
86		Alpha-3	7983	148	94	652	374	99	47	478	505	OP712081
87		Alpha-3	7999	148	96	650	375	100	47	472	504	OP711973
88		Gamma-5	7328	142	98	607	400	194		521	510	NC_010329
89		Alpha-3	8074	150	98	654	377		48	474	502	OP971058
90		Alpha-14	8033	148	98	646	380	102		467	505	NC_004104
91		Alpha-8	7950	155	98	653	370		43	460	502	OP971097
92		Beta-4	7461	138	91	612	477	231		523	512	NC_004500
93		Beta-1	7450	146	97	603	476	220		517	513	AY382778
94		Alpha-2	7881	148	87	683	378	100		459	532 (504)	AJ620211
95		Gamma-1	7337	140	99	601	401	167		524	515	AJ620210
96		Beta-5	7438	225	99	608	468	212		521	512	AY382779
97		Alpha-7	7843	158	106	650	367	88	71	460	533	EF202168
98		Beta-1	7466	153	95	610	474	272		522	512	FM955837
99		Beta-1	7698	155	103	604	504			518	543	FM955838
100		Beta-2	7380	152	100	605	440	184		529	542	FM955839
101		Gamma-6	7258		98	636	393	118		531	515	OP712017
102		Alpha-3	8056	147	96	651	380	120	47	475	503	KU298947
103		Gamma-6	7263		100	621	391	120		525	515	LR861918
104		Beta-2	7386	138	104	610	446	210		520	528	FM955840
105		Beta-1	7667	155	101	603	504			518	520	FM955841
106		Alpha-14	8035	163	95	646	383	131		466	567	DQ080082
107		Beta-2	7562	140	102	607	465	210		519	507	EF422221
108		Gamma-6	7149		99	626	390			517	513	NC_012213
109		Gamma-7	7346	140	96	616	406	152		521	517	NC_012485
110		Beta-2	7423	141	94	607	454	198		537	506	EU410348
111		Beta-2	7384	141	92	579	450	194		534	506	EU410349
112		Gamma-8	7227	139	97	600	392	124		526	526	NC_012486
113		Beta-2	7412	149	92	606	457	215		534	508	FM955842
114		Alpha-3	8069	148	94	625	381	108		475	507	GQ244463
115		Beta-3	7476	138	100	614	481	210		518	512	FJ947080

116	Gamma-9	7184	141	98	598	395		522	523	FJ804072
117	Alpha-2	7895	147	87	679	382		463	533	GQ246950
118	Beta-1	7597	136	99	606	506		517	511	GQ246951
119	Gamma-8	7251	139	97	597	392	133	527	527	GQ845441
120	Beta-2	7304	164	97	604	430	175	519	506	FN598907
121	Gamma-10	7342	143	98	609	390	125	522	519	NC_014185
122	Beta-2	7397	164	97	604	430	175	519	506	GQ845444
123	Gamma-7	7329	137	94	611	401	128	524	520	GQ845445
124	Beta-1	7489	136	97	607	481	211	528	511	GQ845446
125	Alpha-2	7809	148	87	662	377	98	475	531 (503)	FN547152
126	Gamma-11	7326	143	100	607	395	174	499	523	NC_016157
127	Gamma-12	7187	139	98	604	403		509	509	NC_014469
128	Gamma-13	7259	143	96	605	399	163	511	504	NC_014952
129	Gamma-9	7219	152	97	601	386	165	505	517	NC_014953
130	Gamma-10	7388	143	98	609	394	133	525	520	GU117630
131	Gamma-14	7182	141	94	663	395	130	511	513	NC_014954
132	Gamma-12	7125	139	92	399	395	121	510	508	NC_014955
133	Gamma-10	7358	143	99	609	391	127	524	518	GU117633
134	Gamma-7	7309	137	91	602	416	161	514	516	NC_014956
135	Gamma-15	7293	138	95	601	403	148	512	515	NC_017993
136	Gamma-11	7319	142	98	603	392	147	503	523	NC_017994
137	Gamma-16	7236	142	99	610	403	132	509	516	NC_017995
138	Gamma-7	7353	137	93	616	404	138	519	514	HM999990
139	Gamma-7	7360	136	93	617	405	139	518	518	HM999991
140	Gamma-11	7341	143	99	605	392	125	498	528	NC_017996
141	Gamma-11	7384	144	98	604	392	147	500	523	HM999993
142	Gamma-10	7374	143	97	609	397	129	524	520	HM999994
143	Beta-1	7715	157	103	605	508	243	518	516	HM999995
144	Gamma-17	7271	140	99	599	387	150	512	517	NC_017997
145	Beta-2	7375	141	96	606	433	197	532	507	HM999997
146	Gamma-15	7265	140	97	606	402	147	509	515	HM999998
147	Gamma-8	7224	139	96	601	395	162	521	521	HM999999
148	Gamma-12	7164	138	93	602	396	124	503	512	Gu129016
149	Gamma-7	7333	136	94	612	406	174	521	516	GU117629
150	Beta-5	7436	136	100	605	470	214	518	510	FN677755
151	Beta-2	7386	165	98	608	437	214	524	511	FN677756
152	Beta-1	7480	136	97	607	478		520	511	JF304768
153	Gamma-13	7240	143	96	605	400	144	511	511	JN171845
154	Gamma-11	7286	144	100	606	392	148	506	526	NC_021483
155	Alpha-7	7352	145	94	606	405	164	518	514	JF906559
156	Gamma-18	7329	140	97	608	399	201	551	509	NC_033781
157	Gamma-12	7154	138	93	601	400		506	510	KT698166
158	Gamma-12	7192	140	95	600	391		520	512	KT698168
159	Beta-2	7443	141	92	606	459	203	508	531	HE963025
160	Alpha-2	7779	144	91	660	371	120	474	531	AB745694
161	Gamma-19	7238	140	97	604	392		513	507	NC_038522
162	Gamma-19	7214	139	101	607	389		506	506	JX413108
163	Gamma-20	7233	142	99	607	406		516	507	NC_028125
164	Gamma-8	7233	139	97	600	395	163	524	525	JX413106
165	Gamma-12	7129	139	93	608	396		480	506	JX444072
166	Gamma-19	7212	139	101	607	389		510	506	NC_019023
167	Gamma-21	7228	139	97	604	401		518	510	NC_022892
168	Gamma-8	7204	139	97	600	392	160	520	523	KC862317
169	Gamma-11	7252	143	99	603	390	172	496	525	JX413105
170	Gamma-7	7417	145	94	617	404		522	518	JX413110
171	Gamma-11	7261	143	98	604	390	172	496	525	KF006398
172	Gamma-22	7203	183	98	600	395	139	517	516	NC_038523
173	Gamma-1	7293	140	97	603	391		527	513	KF006400

174	Beta-2	7359	141	95	601	448	192	526	507	HF930491
175	Gamma-23	7226	147	97	607	403	166	513	504	NC_038524
176	Gamma-8	7226	139	96	598	395		522	523	KR816167
177	Alpha-11	7933	154	100	656	364		471	503	KR816168
178	Gamma-24	7314	138	95	608	401	166	531	511	NC_023891
179	Gamma-15	7228	138	95	602	399		511	512	NC_022095
180	Gamma-10	7356	143	98	609	390	125	524	519	KC108722
181	Gamma-11	7240	141	97	604	388		500	524	KR816169
182	Beta-2	7416	141	93	605	457		533	507	KR816170
183	Gamma-20	7286	142	99	603	404		518	510	KR816171
184	Gamma-25	7324	143	97	603	405		524	521	NC_038914
185	Beta-5	7444	136	99	611	465		521	512	KR816172
186	Gamma-7	7389	137	94	614	401		523	521	KR816173
187	Gamma-27	7268	139	96	603	394		527	514	NC_039086
188	Gamma-3	7185	141	93	599	393		506	515	KR816175
189	Gamma-7	7318	143	94	612	401		523	517	KR816176
190	Gamma-24*	7275	147	95	607	395		531	511	KR816177
191	Gamma-10	7364	143	98	609	394		524	521	KR816178
192	Gamma-15	7244	139	98	604	397		517	513	KR816179
193	Gamma-7	7348	140	93	609	408		522	518	KR816180
194	Gamma-20*	7260	142	99	607	403		518	507	KR816181
195	Beta-1	7569	136	99	606	501		516	511	KR816182
196	Beta-1	7500	136	97	606	483		519	511	KR816183
197	Gamma-24	7278	147	94	607	392	158	532	515	KM085343
198	Beta-2*							510		MG921179
199	Gamma-12	7184	139	97	598	398		509	507	KJ913662
200	Gamma-2	7137	139	93	598	401		502	514	KP692114
201	Gamma-27	7221	140	98	608	418	182	517	508	NC_027528
202	Gamma-11	7344	143	99	605	392	122	498	528	KP692116
203	Gamma-7*	7372	138	95	613	406	70	532	516	MG921180
204	Mu-3	7227	136	92	615	388	131	503	508	NC_038525
205	Gamma-1	7298	140	97	603	392		527	515	KT698167
206**	Beta-1	7731	157	103	606	511	242	532	516	U85660
207	Gamma-15*	7247	139	97	601	354		459	514	MK645900
208	Gamma-24*	7283	148	87	607	400		527	511	MK645901
209	Beta-2	7399	140	102	607	457	202	519	507	KY242583
210	Beta-12	7134	139	92	599	363		513	508	MH460956
211	Gamma-8*	7253	139	96	604	396	146	532	516	MF509816
212	Gamma-17*	7208	148	99	599	387	116	508	517	MF509817
213	Gamma-13*	7096	144	97	603	391	158	497	508	MF509818
214	Gamma-6*	7357		100	634	388	117	537	511	MF509819
215	Gamma-9*	7186	145	97	603	383	163	503	516	MF509820
216	Gamma-9*	7233	152	97	603	390	170	505	517	MF509821
219	Gamma-13*	7108	144	97	603	390	154	497	508	MH172376
220	Gamma-17*	7381	140	99	602	388	150	533	514	MH172377
221	Gamma-10*	7326	143	97	610	394	126	523	520	MH172378
222	Gamma-19*	7275	140	96	600	398	119	510	512	MH172379
223**	Gamma-22*	7223	140	99	598	399	138	516	520	MG063749
224**	Gamma-8*	7233	139	96	598	397	123	519	513	MF356498
225**	Gamma-7*	7320	137	94	610	399	126	523	520	MG520499
226**	Gamma-6*	7313		97	636	392	114	531	515	MG813996
227**	Beta-2*	7441	140	94	610	454	198	538	507	MK080568
228	Gamma-27*	7277	140	97	605	420	165	517	508	ON482334
229	Gamma-7*	7319	137	91	602	417	162	514	483	MW535770
230	Gamma-11	7300	140	98	601	408		509	521	OQ915151
231**	Gamma-10*	7360	143	99	609	376		524	518	OP577477
XS2	Alpha-2	7830	148	87	660	378		474	504	OL817334
